# Potential targeted therapy and diagnosis based on novel insight into growth factors, receptors, and downstream effectors in acute kidney injury and acute kidney injury-chronic kidney disease progression

**DOI:** 10.1038/s41392-020-0106-1

**Published:** 2020-02-14

**Authors:** Li Gao, Xiang Zhong, Juan Jin, Jun Li, Xiao-ming Meng

**Affiliations:** 1 The Key Laboratory of Major Autoimmune Diseases, Anhui Institute of Innovative Drugs, School of Pharmacy, Anhui Medical University, 230032 Hefei, China; 2 Department of Nephrology, Sichuan Academy of Medical Sciences & Sichuan Provincial People’s Hospital, School of Medicine, University of Electronic Science and Technology of China, 610072 Chengdu, Sichuan China; 3 Department of Pharmacology, Key Laboratory of Anti-inflammatory and Immunopharmacology, Ministry of Education, Anhui Medical University, 230032 Hefei, China

**Keywords:** Predictive markers, Prognostic markers

## Abstract

Acute kidney injury (AKI) is defined as a rapid decline in renal function and is characterized by excessive renal inflammation and programmed death of resident cells. AKI shows high morbidity and mortality, and severe or repeated AKI can transition to chronic kidney disease (CKD) or even end-stage renal disease (ESRD); however, very few effective and specific therapies are available, except for supportive treatment. Growth factors, such as epidermal growth factor (EGF), insulin-like growth factor (IGF), and transforming growth factor-β (TGF-β), are significantly altered in AKI models and have been suggested to play critical roles in the repair process of AKI because of their roles in cell regeneration and renal repair. In recent years, a series of studies have shown evidence that growth factors, receptors, and downstream effectors may be highly involved in the mechanism of AKI and may function in the early stage of AKI in response to stimuli by regulating inflammation and programmed cell death. Moreover, certain growth factors or correlated proteins act as biomarkers for AKI due to their sensitivity and specificity. Furthermore, growth factors originating from mesenchymal stem cells (MSCs) via paracrine signaling or extracellular vesicles recruit leukocytes or repair intrinsic cells and may participate in AKI repair or the AKI-CKD transition. In addition, growth factor-modified MSCs show superior therapeutic potential compared to that of unmodified controls. In this review, we summarized the current therapeutic and diagnostic strategies targeting growth factors to treat AKI in clinical trials. We also evaluated the possibilities of other growth factor-correlated molecules as therapeutic targets in the treatment of AKI and the AKI-CKD transition.

## Introduction

Acute kidney injury (AKI) is a clinical syndrome with acute renal dysfunction. The major causes of AKI include ischemic reperfusion, drug toxicity, and sepsis.^1^ The common pathological feature of AKI is damage to tubular epithelial cells (TECs), accompanied by endothelial damage and accumulation of inflammatory cells.^2–4^ AKI shows high morbidity and mortality, and severe or repeated AKI may progress to chronic kidney disease (CKD) or even end-stage renal disease (ESRD).^5^ Unfortunately, effective and specific therapies are unavailable, except for supportive management.^1,6,7^

In the last century, growth factors such as epidermal growth factor (EGF), insulin-like growth factor (IGF), and fibroblast growth factor (FGF) have been widely investigated as an interesting research area since they are significantly dysregulated and dysfunctional in different AKI models^8^ (Table 1). Evidence has shown that the administration of these growth factors promotes renal repair and restores renal function in animals; however, treatment with growth factors has not been used clinically.^9^ With the rapid progress in research technology, growth factors, receptors, and downstream effectors have been found to be highly involved in the mechanism of AKI, including the regulation of inflammation, programmed cell death, necrosis, cell proliferation, and dedifferentiation.^10^ Moreover, certain growth factors or correlated proteins, such as IGF binding protein (IGFBP)-7 and FGF-23, can serve as biomarkers for AKI due to their sensitivity and specificity.^11^ Paracrine or extracellular vesicle-delivered growth factors, such as hepatocyte growth factor (HGF) or vascular endothelial growth factor (VEGF), are major mechanisms by which mesenchymal stem cells (MSCs) exert therapeutic effects on renal injury. Growth factor-modified MSCs show superior therapeutic effects in AKI treatment.^12^ Therefore, the current review focused on summarizing the use of various growth factors as biomarkers for predicting AKI and interpreting their functions and the mechanisms underlying their roles in both renal injury and renal repair in AKI. We also evaluated the current growth factor-targeted therapy or diagnosis in clinical trials and analyzed the limitations of growth factors in clinical treatment. These findings may add new information to the search for a target and prediction of AKI and AKI-CKD progression.

**Table 1:** Growth factors may contribute to different types of AKI.

Model of AKI	Growth factor
Ischemia-reperfusion Injury	BMP-7/EGF/FGF-2/HGF/ IGF-1/TGF-β1/VEGF/PDGF
Folic acid-induced AKI	BMP-7/EGF/FGF-23/HGF/ TGF-β1/VEGF/IGF-1
Cisplatin-induced AKI	BMP-7/TGF-β1/VEGF/ FGF-21/FGF-10/EGF/IGF-1/HGF
Lipopolysaccharide-induced AKI	BMP-7/FGF-2/TGF-β1/HGF/EGF
Mercuric chloride-induced AKI	EGF/IGF-1
Glycerol-induced AKI	HGF/TGF-β1
Colistin-induced AKI	TGF-β1/EGF
Gentamicin-induced AKI	IGF-1/TGF-β1/EGF/PDGF/VEGF

## Growth factors in AKI

### Bone morphogenetic proteins in AKI

**Fig. 1:**
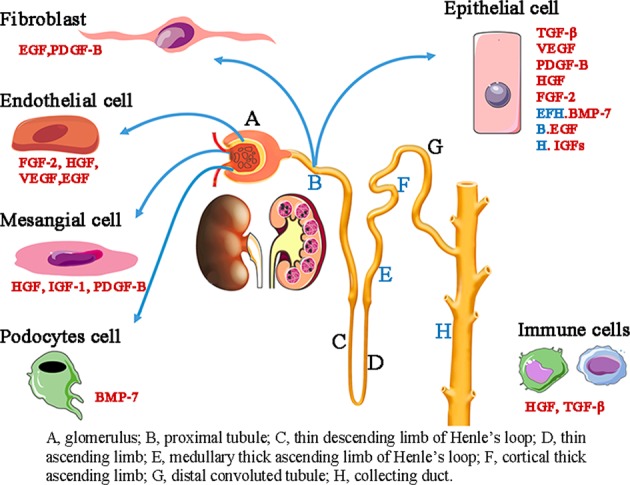
Localization of growth factor expression in the kidney. In the glomerulus, endothelial cells mainly secrete FGF-2, HGF, and VEGF, mesangial cells secrete HGF, IGF-1, and PDGF-B, and podocytes are the major source of BMP-7. In renal tubules, growth factors are primarily expressed in fibroblasts and epithelial cells. Fibroblasts express and secrete EGF and PDGF-B, and epithelial cells secrete TGF-β, VEGF, PDGF-B, HGF, and FGF-2. Specifically, BMP-7 is only detected in thick ascending limb and collecting duct epithelial cells. EGF is expressed in the proximal tubule, and IGFs are secreted in the collecting duct. Infiltrating inflammatory cells, such as macrophages, are the key source of HGF, TGF-β, and PDGF-B.

Bone morphogenetic proteins (BMPs) are conserved signaling molecules that belong to the transforming growth factor-β1 (TGF-β) superfamily. Structurally, BMPs and some TGF-β family members act as monomeric prepro-forms, including signal sequences, long latency-associated peptides (LAPs), and mature cytokines. These precursor dimers are cleaved by an enzyme at R-X-X-R proteolytic processing sites, which release the biologically active domain. There is the highest degree of similarity (~40–70%) at the carboxy-terminal regions among mature peptides that are the biologically active form of BMP-7 and TGF-β1.^13^ To date, no less than fifteen BMPs have been identified.^14^ Recently, more attention has been focused on BMP-7, which is also known as osteogenic protein-1 (OP-1), for its protective role in acute and chronic kidney diseases. In the adult kidney, BMP-7 was detected specifically in the collecting tubule, the thick ascending limb, and podocytes^15^ (Fig. 1); however, BMP-7 expression is significantly reduced in different kidney diseases, including AKI.^16^

The first evidence regarding the protective role of BMP-7 in AKI was found in a study that indicated that OP-1 injection preserved kidney function and increased the survival rate after ischemic AKI through several mechanisms. These mechanisms included reducing apoptosis and necrosis of tubular epithelial cells, suppressing inflammation by limiting neutrophil infiltration and the level of intercellular adhesive molecules, and maintaining the vascular smooth muscle cell phenotype in pericellular capillaries.^17^ The anti-inflammatory effect of BMP-7 was also highlighted in another study that indicated that BMP-7 regulated the expression of chemokines, cytokines, and hemodynamic genes (vasoactive genes) in proximal tubule cells.^15^ By generating tubular-specific BMP receptor 1A knockout mice, a recent study showed that BMP-7/Smad1/5/8 signaling accelerated tubular regeneration by targeting the inhibition of DNA-binding (ID) proteins (Id1, Id2, and Id4), thereby mediating recovery after AKI and preventing fibrosis.^18^

Evidence shows that BMP-7 acts as a key target in the pathological process of AKI. By modifying ligand-receptor interactions to enhance BMP-7 and suppress TGF-β signaling, Kielin/chordin-like protein (KCP) is capable of halting folic acid-induced AKI by decreasing mortality while enhancing the recovery of renal function.^19^ Signal peptide-CUB epithelial growth factor domain-containing protein 1 (SCUBE1) directly binds to the BMP-7 ligand and stimulates Smad1/5/8 phosphorylation, thereby accelerating tubular cell proliferation and re-epithelization after renal ischemia-reperfusion injury (IRI).^20^ Knockout of uterine sensitization-associated gene-1 (USAG)-1, the most abundant BMP antagonist in the kidney, significantly prolonged survival, and preserved renal function in the AKI model, whereas the administration of neutralizing antibodies against BMP-7 abrogated the renoprotective effect of USAG-1 deficiency, further indicating that BMPs are promising therapeutic targets in AKI treatment.^21^ In addition, MyoR inhibits cisplatin-induced apoptosis and deterioration of renal function by targeting BMP-7.^22^ Propofol, a sedative, suppresses oxidative stress in sepsis-induced AKI by upregulating BMP-7.^23^ Additionally, epigenetic modification of BMP-7 plays a critical role in AKI progression. In AKI models induced by both ischemic reperfusion and sepsis, dexmedetomidine (DEX), an α (2)-adrenoceptor (α(2)-AR) agonist, protects against renal injury by restoring BMP-7 levels via a histone deacetylase 5 (HDAC5)-dependent mechanism.^24,25^ Furthermore, we recently showed that the HDAC inhibitors trichostatin A (TSA) or valproic acid (VPA) attenuated cisplatin-induced renal tubular epithelial cell apoptosis by restoring BMP-7 expression via targeting HDAC2.^26^

The protective effect of BMPs in AKI is also attributed to their protective role in endothelial cells. A study found that BMP-5 promoted the migration and survival of early endothelial outgrowth cells (eEOCs), thereby improving renal function in the short term.^27^ Collectively, the therapeutic effect of BMP-7 may be due to its anti-inflammatory, antiapoptotic, and proliferative effects. However, the function of other members of the BMP family in AKI is still unknown and needs to be further determined.

### EGF and the EGF receptor in AKI

The EGF-related peptide growth factor family consists of groups of ligands. The first group includes EGF, transforming growth factor-α (TGF-α), and amphiregulin. These factors work by specifically binding to EGF receptor (EGFR). Members of the second group, including heparin-binding EGF (HB-EGF) and betacellulin, bind to both EGFR and ErbB4.^28^ Activation of EGF/EGFR triggers downstream intracellular pathways, including MAP kinase, JAK/STAT, and PI3K/AKT, to control cell apoptosis, proliferation, and differentiation.

In the kidney, EGF is highly expressed in renal proximal tubule epithelial cells (RPTCs) and transiently decreases after IRI^29^ (Fig. 1). Clinical evidence shows that urinary human EGF (hEGF) levels are largely downregulated in patients with AKI compared to those of control subjects.^30^ A study showed that the administration of exogenous EGF increased the DNA replication and recovery of renal function in IRI.^31^ EGF also attenuates mercuric chloride (HgCl_2_)-induced tubular necrosis by stimulating the regeneration of resident cells rather than bone marrow-derived cells.^32^

EGFR function has attracted more attention in recent years. EGFR is widely expressed in mammalian kidneys, with high levels of expression in RPTCs and interstitial fibroblasts. It is a transmembrane protein with intrinsic tyrosine kinase activity and can be activated by several ligands, such as EGF, TGF-β1 and IGF.^28,33^ Activation of EGFR can be detected 5–30 min after reperfusion, accompanied by generation of superoxide anion/hydrogen peroxide and a reduction in EGF. This finding indicates that early activation of EGFR may not be EGF-dependent.^29^ Functional studies showed that conditional deletion of EGFR from RPTCs or treatment with an EGFR tyrosine kinase inhibitor (erlotinib) delayed renal function recovery on day 6 after IRI, but activation of EGFR with exogenous EGF or HB-EGF accelerated renal repair.^34^ Furthermore, a recent study identified that EGFR promoted the dedifferentiation and proliferation of surviving RPTCs by activating Yes-associated protein (YAP) and transcriptional coactivator with PDZ binding motif (TAZ).^35^ Strikingly, deletion of ErbB4, a type I transmembrane receptor tyrosine kinase of the EGFR superfamily, accelerated cell proliferation and unbalanced cell apoptosis, which was related to the activation of YAP, resulting in renal function deterioration and fibrosis following ischemic injury. This finding was further confirmed in other renal fibrosis models, such as polycystic kidney disease and UUO nephropathy.^36,37^ As mentioned previously, activating EGF/EGFR signaling appears to be a promising strategy for treating AKI and recovery after AKI.^38^ However, it is noteworthy that sustained activation of EGFR is associated with cell cycle arrest at the G2/M phase, leading to renal fibrogenesis after AKI.^39,40^ Therefore, exogenous EGF or HB-EGF may not be suitable for long-term treatment. Consistently, functional inactivation of EGFR by overexpression of dominant-negative EGFR in RPTCs decreases tubulointerstitial lesions after renal injury.^41^ These findings indicate that EGFR may function as a double-edged sword by regulating both repair and fibrosis, which may be determined by the degree and duration of EGFR activation in response to renal injury.^39^

### FGF in AKI

Mammalian fibroblast growth factor signaling involves interactions between 18 FGF ligands and 4 FGF receptors (FGFR1–4).^42^ Some FGFs, such as FGF-2 and FGF-23, play specific roles in mediating or predicting AKI.

FGF-2, also called basic fibroblast growth factor (bFGF), is a well-characterized survival factor for both endothelial cells and epithelial cells^43^ (Fig. 1). Administration of bFGF induces an early repair process after ischemic AKI by inducing various morphogens that are involved in renal repair, such as FGF-2 itself, HGF, BMP-7 and VEGF.^44,45^ This observation was further confirmed by a recent study that found that FGF-2 protected against mitochondrial damage and the HMGB1-mediated inflammatory response induced by IRI.^46^ However, the function of FGF-2 is still controversial. Other studies have shown that increased circulating FGF-2 levels fails to improve the outcome of lipopolysaccharide (LPS)-induced AKI but leads to further renal damage because circulating FGF-2 may predispose endothelial cells to undergo apoptosis in response to LPS or induce inflammatory changes.^43^ This was further confirmed by high serum FGF-2 levels in children with sepsis who were at a high risk of developing AKI. This discrepancy may be explained by the difference in AKI insults.

As a novel predictive and prognostic biomarker for AKI, FGF-23 has recently been widely investigated in different types of animal models and AKI patients.^11^ FGF-23, induced by multiple factors such as IRI, folic acid, and rhabdomyolysis, is significantly increased in AKI patients and murine models.^11,47,48^ Clinical evidence confirmed that FGF-23 levels significantly increase in infants, children, adults, and older individuals suffering AKI.^49–53^ FGF-23 is not only an early prognostic marker for cardiac surgery-associated AKI and intensive care unit (ICU)-associated AKI but also serves as a prognostic marker for adverse outcomes in patients with established AKI.^11^ Several studies have revealed the mechanisms underlying the upregulation of FGF-23 in AKI. A recent study showed that hyper-IL-6 (HIL-6) activates the FGF-23 promoter by STAT3 phosphorylation and increases circulating FGF-23 in both AKI and CKD.^54^ Activation of FGF receptor 1 (FGFR1) further increases FGF-23 synthesis in folic acid-induced AKI.^55^ Moreover, decreased FGF-23 clearance in AKI also contributes to high circulating levels of FGF-23.^11^ However, whether FGF-23 plays a functional role in mediating AKI is an important topic and remains to be explored.

The participation of other FGF ligands or receptors has also been determined. Evidence shows that inhibiting nitric oxide synthase with Nw-nitro-l-arginine (L-NNA) abolishes the suppressive effects of FGF-1 on neutrophil infiltration, indicating that nitric oxide may be involved in the anti-inflammatory effects of FGF-1.^56^ FGF-10 works by binding to the high-affinity receptor FGFR2-IIIb splicing isoform and protects against kidney IRI by inhibiting excessive autophagy and the inflammatory response.^57^ FGF-21, a key regulator of the energy metabolic balance and cell stress responses, is induced in cisplatin nephropathy. FGF-21 knockdown accelerates cisplatin-induced tubular cell injury via p53-dependent mechanisms, but this effect is attenuated by supplementation with recombinant FGF-21.^58^ Additionally, a study showed that bFGFR2 knockdown prevented the repair process and induced a fibrotic response after ischemic injury, indicating the therapeutic potential of bFGFR2 in AKI.^59^

### Hepatocyte growth factor and c-met in AKI

HGF was originally isolated as a potent mitogen for hepatocytes that binds to the c-met receptor and stimulates its transactivation. HGF exerts multiple effects on tubular repair and regeneration in the kidney.^60–62^ Epithelial cells, mesangial cells, endothelial cells, and macrophages are the major origins of renal HGF^62^ (Fig. 1). In the early phase of AKI, HGF, and c-met mRNA significantly increase; however, the total protein level of HGF in the kidney is downregulated 24 h post injury.^60^ Evidence shows that previous partial hepatectomy-induced HGF overexpression attenuates tubular apoptosis and necrosis.^63^ Furthermore, HGF gene therapy reduces renal failure and mortality by attenuating tubulointerstitial damage, proinflammatory cytokine production, necrosis, and hemodynamic deterioration.^64,65^ Previous studies showed that human umbilical cord-derived MSC (hucMSC) transplantation improved renal function in ischemia/reperfusion-induced AKI rats, and HGF-modified hucMSCs showed high efficiency in treating AKI via antiapoptotic and anti-inflammatory mechanisms.^66^ Additionally, evidence showed that HGF-transgenic mesothelial cell sheet transplantation supports renal recovery and attenuates fibrosis in AKI murine models.^67,68^ It is noteworthy that the balance between HGF and TGF-β signaling at the initial stage of IRI facilitates the acute repair response, but the balance switches to TGF-β signaling during abnormal repair and fibrogenesis.^69^ In addition, HGF is highly correlated with active β-catenin in fibroblasts. β-catenin deficiency in renal fibroblasts induces HGF expression and activates tyrosine phosphorylation of the c-met receptor after IRI, thereby promoting cell proliferation and renal repair.^70^ In injured kidneys, proHGF is processed and cleaved to form mature HGF that binds to the c-met receptor.^62^ Conditional knockout of c-met in renal tubules exacerbates renal injury and inhibits renal regeneration after AKI. This indicates that tubule-specific c-met signaling plays an essential role in renal protection due to its proliferative, antiapoptotic, and anti-inflammatory properties.^71–73^ Consistently, HGF/c-met attenuates renal injury and inflammation while accelerating repair after glycerol-induced AKI.^74^

### IGF and IGFBPs in AKI

IGF, a peptide growth factor that is secreted by the collecting duct of the adult kidney, binds with IGF1R and phosphorylates insulin receptor substrate proteins, thereby initiating downstream pathways, including PI3K-Akt-mTOR, to participate in the regulation of cell proliferation and apoptosis^75,76^ (Fig. 1). Infusion of IGF-1 improves hemodynamic parameters, such as renal plasma flow (RPF), inulin clearances (GFR), and renal vascular resistance (RVR), in fasted rats.^77^ Previous studies have shown that IGF signaling is highly involved in kidney development and different types of kidney diseases, including AKI.^78,79^ However, the function of IGF in AKI is still controversial. IGF-1 decreases following ischemic injury, and treatment with exogenous IGF-1 accelerates recovery by limiting cell apoptosis and promoting cell proliferation.^80,81^ These findings were further confirmed by a study indicating that administration of rhIGF-1 2 h post injury suppresses the renal inflammatory response and upregulates EGF levels.^82^ IGF-1 also promotes tubular regeneration after AKI by transactivating EGFR.^83^ In contrast, it is unfortunate that data from a clinical trial showed less salutary results for IGF-1 treatment,^84,85^ because administration of IGF-1 induced an inflammatory response, especially neutrophil accumulation, in rats with AKI, and this may lead to a higher mortality risk in patients.^86,87^ In addition, induction of the fibrotic response in mesangial cells may be another reason for the failure of IGF-1 treatment.^88^ Although IGF-1-based AKI therapy is disappointing, serum IGF-1 appears to be a potential biomarker because a reduced level of serum IGF is clearly correlated with increased mortality and the nutritional status of patients. The serum stability and short half-life of IGF-1 make it a suitable candidate as an early and sensitive biomarker for AKI mortality in intensive care units.^89^ In addition to IGF ligands, receptors, and insulin, a family of high-affinity IGFBPs has been identified in the IGF system and has gained more attention. These factors primarily antagonize IGF actions and may serve as biomarkers for AKI.^90^ Among these IGFBPs, IGFBP-7 is well studied, and emerging evidence shows that urinary IGFBP-7 and tissue inhibitor of metalloproteinase-2 (TIMP-2) can be applied as early diagnostic biomarkers for AKI following cardiac surgery,^91^ sepsis,^92^ and other renal insults of varied etiology.^93–96^ These factors appear to be ideal biomarkers for moderate and severe AKI, and the US Food and Drug Administration already permitted marketing of NephroCheck® (Astute Medical) to detect urinary [TIMP-2]*[IGFBP-7] in critically ill patients in 2014.^90,95,97^ However, it is noteworthy that the kinetics of urinary TIMP-2 and IGFBP-7 do not match the exposure of radiocontrast in patients suffering from stage 2–3 AKI.^98^

### TGF-β in AKI

TGF-β exerts multiple biological functions in renal diseases by binding to its receptors and activating downstream Smad and non-Smad pathways, and renal TGF-β mainly originates from epithelial cells, leukocytes, or the circulation^99,100^ (Fig. 1). TGF-β1 is a well-recognized profibrotic factor.^101–103^ Activation of TGF-β/Smad signaling is detected in AKI models induced by different types of insults, such as IRI.^104^ In the IRI model, the level of TGF-β1 is increased by 1.5-fold at 12 h and more than 3-fold at 24 h and is sustained at a high level until 14 days,^105^ which was confirmed by our recent study showing that the production of TGF-β1 was significantly induced in cisplatin nephropathy.^106^ However, the exact role of TGF-β in AKI is not fully understood.

Several studies have provided evidence that TGF-β1 may be protective in AKI. It has been reported that a deficiency in TGF-β1 in mice increases renal damage and deteriorates renal function,^107^ and this was further confirmed by another study showing that sevoflurane protects against IRI-induced renal injury.^108^ Additionally, a recent study showed that TGF-β-induced CD4 + Foxp3 + Tregs prevented antibody-mediated acute renal allograft injury by targeting multiple effectors.^109^ However, other studies have shown controversial data that TGF-β1 is possibly detrimental in AKI. In the IRI rat model, blockade of TGF-β1 signaling with anti-TGF-β antibodies attenuates renal hypertrophy and interstitial cellularity and has a beneficial effect on microvascular structure but fails to accelerate the recovery of renal function.^110^ This finding was further confirmed by a recent study showing that SB4315432, a TGF-β1 receptor I inhibitor, decreased Nox4 levels and cell injury following colistin exposure.^111^ In addition, overexpression of type I TGF-β receptors specifically in tubular epithelial cells is sufficient to induce acute tubular injury and renal inflammation, which partly depends on mitochondrial-derived ROS.^112^ Consistently, conditional knockout of type II receptors from tubular epithelial cells blocks hydrogen peroxide–induced apoptosis, at least partly, through a Smad-dependent mechanism.^113^ Some studies revealed the potential function of downstream Smads in AKI. Global knockout of Smad3 protects against ischemic AKI by reducing IL-6 production.^114^ Moreover, Smad3 binds directly to p27 and inhibits the CDK2/cyclin E complex, thereby promoting AKI.^115^ As an inhibitory Smad, Smad7 protects against AKI by rescuing tubular epithelial cells from Smad3-mediated G1 cell cycle arrest.^116^ The function of Smad2 in AKI has drawn attention. A recent study showed that the activation of Smad2 is highly correlated with AKI progression.^109^ Our group further identified that conditional knockout of Smad2 protects against AKI by alleviating cell necroptosis, apoptosis and inflammation via the Smad/p53 interaction.^106^ Interestingly, we previously reported that Smad2 protects against renal fibrosis by suppressing Smad3 signaling;^117^ however, Smad2 and Smad3 are both detrimental in the progression of AKI, which indicates that the functional interaction between Smad2 and Smad3 might be distinct in different conditions; this needs to be further determined in future studies.^106^

It is noteworthy that TGF-β/Smads play a predominant role in the progression of AKI to CKD.^118^ In the tubular injury phase, proximal tubular cells dedifferentiate and proliferate to replace lost epithelial cells. However, when the insult is severe and unresolvable, some cells fail to redifferentiate and continue to produce growth factors such as TGF-β, finally leading to renal fibrosis.^119^ Additionally, a recent study showed that TGF-βRII deletion in macrophages prevents tubulointerstitial fibrosis following severe ischemic renal injury by abrogating TGF-β-dependent chemoattraction of macrophages.^118^ Collectively, the functions of TGF-β/Smads may vary according to their activation level, disease stages, and types of AKI models, which need to be further validated. Exploring the detailed function of TGF-β and downstream Smads may help us to better understand the pathological mechanisms of AKI and its progression to CKD.

### VEGF in AKI

In the kidney, VEGF is mainly expressed in epithelial and endothelial cells (Fig. 1). Five isoforms of amino acids 121, 145, 165, 189, and 206 are produced through alternative splicing of VEGF mRNA. These amino acids bind to VEGFR-1 (flt-1), VEGFR-2 (flk-1), or VEGFR-3 to perform biological functions. In response to ischemic AKI insults, VEGFR-2 is upregulated in kidney tissues, although VEGF mRNA and protein levels are not increased, suggesting the possibility for exogenous VEGF treatment.^120–122^ A study showed that treatment with VEGF-121 protects against renal microvessel structure and prevents the AKI-CKD transition in response to increased sodium intake.^123^ Mechanistically, VEGF promotes renal repair following AKI by directly mediating mitogenic and antiapoptotic effects on TECs.^124^ In addition, VEGF expression stabilizes microvascular density, diminishes capillary rarefaction, and improves renal perfusion, which decreases chronic hypoxia and hemodynamics in ischemic AKI.^125,126^ Of note, the transcriptional regulation of VEGF has drawn increasing attention. As a key transcription factor, hypoxia inducible factor-1 (HIF-1) induces VEGF production to protect against hypoxic renal injury in the acute hypoxia phase of the ischemic AKI model.^127^ Preischemic targeting of HIF prolyl hydroxylation attenuates AKI and prevents AKI-CKD progression.^128^ However, HIF-1-induced overproduction of several growth factors (such as VEGF and connective tissue growth factor (CTGF)) contribute to renal fibrosis in chronic hypoxia conditions.^129,130^ Thus, the disease condition might be critical when HIF-1/VEGF-targeted therapy is applied.

### Platelet-derived growth factor in AKI

Platelet-derived growth factors (PDGFs) consist of five dimers termed PDGF-AA, -AB, -BB, -CC, and -DD, and they bind and activate PDGF receptors (PDGFR-αα, -αβ, and -ββ) with distinct binding affinities.^131^ PDGFs are secreted by injured epithelial cells after AKI, and other cells involved in the progression of CKD also secrete PDGFs, including mesangial cells, fibroblasts, and pericytes^131^ (Fig. 1). Similarly, PDGF receptors are predominantly expressed on mesenchymal cells.^132^ In the early phase of IRI, PDGF-B/PDGFR is expressed in the S3 segments of the proximal tubule. This is related to proliferation activated by Src kinase, which induces tubular epithelial cell self-renewal.^133,134^ Concurrently, PDGF-B signaling is highly involved in fibroblast transformation, capillary damage, and rarefaction that result in alterations in renal hemodynamics. This indicates that PDGF contributes to the development of the AKI-CKD transition.^10^ However, the function of PDGF and PDGFR in the AKI-CKD transition, especially in the early stage, should be verified with conditional knockout models.

## Growth factors and the AKI-CKD transition

### Pathophysiology of the AKI-CKD transition

**Fig. 2:**
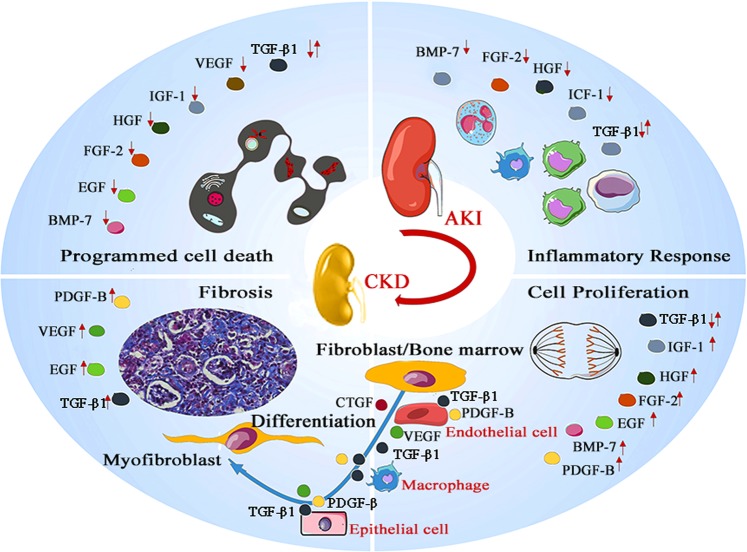
Effect of growth factors on AKI and AKI-CKD progression. Many growth factors, such as BMP-7, EGF, FGF-2, HGF, IGF-1, VEGF, and TGF-β1, are involved in the programmed cell death of endothelial or epithelial cells in the acute injury phase. BMP-7, FGF-2, HGF, TGF-β1, and IGF-1 participate in the regulation of the inflammatory microenvironment that is responsible for cytokine production and immune cell recruitment. TGF-β1 is a double-edged growth factor. In addition, TGF-β1 exerts anti-inflammatory effects, and TGF-β1 overproduction leads to acute tubular injury. After injured epithelial cells fail to regenerate through differentiation, fibrosis is induced as a self-limiting repair process to limit damage. In this stage, overproduction of growth factors such as TGF-β1, PDGF, and FGF induces fibroblast/pericyte proliferation, transdifferentiation of tubular epithelial cells, endothelial cells, and macrophages, and extracellular matrix production, leading to CKD. Concurrently, abnormal synthesis of PDGF-B, VEGF, EGF, and TGF-β1 has a negative impact on endothelial integrity and causes capillary rarefaction, accelerating renal fibrosis.

Accumulating evidence indicates that the severity of AKI and the number of AKI episodes are positively correlated with the subsequent development of CKD.^135^ When renal ischemia, toxic exposure, or obstruction occurs, TECs initiate renal self-renewal, including redifferentiation and proliferation, to replace the injured cells.^136^ Moreover, G2/M phase cell cycle arrest of some TECs results in a failure to regenerate and acquire a profibrotic phenotype, mediating the secretion of fibrotic cytokines such as TGF-β and CTGF, which accelerate the course of interstitial fibrosis, including fibroblast/bone marrow (major precursors of fibroblasts) differentiation or proliferation.^136,137^ Additionally, ischemia and oxidative stress induce endothelial cell apoptosis, which mediates microvasculature rarefaction, causing leakage of large macromolecules that are responsible for inflammatory and profibrotic responses in the interstitium^137^ (Fig. 2). An epidemiological study showed that the incidence of AKI-CKD transition occurs in ~15–20% of 1.5 million AKI survivors per year.^138^ Therefore, the molecular mechanisms underlying the AKI-CKD transition attract much attention. Possible mechanisms contributing to AKI-CKD progression include unresolved renal inflammation, tubular epithelial cell G2/M phase cell cycle arrest, hypoxia, microvascular rarefaction, transdifferentiation, and senescence of resident renal cells, myofibroblast activation, and interstitial fibrosis.^139^

### Growth factors in the AKI-CKD transition

Emerging evidence shows that growth factors are highly involved in the progression of AKI to CKD.^10,140^ EGFR signaling is closely correlated with CKD progression. In a vancomycin-induced AKI mouse model, mutations in EGFR or inactivation of EGFR with gefitinib prevents the AKI-CKD transition via the STAT3/homeodomain interacting protein kinase 2 (HIPK2) axis.^141^ TGF-β is another key mediator that links AKI to CKD, although it has anti-inflammatory effects in certain conditions. TGF-β has multiple effects on renal cells in the AKI stage. For instance, TGF-β induces macrophage chemotaxis to accelerate inflammation and increase apoptosis of tubular epithelial and endothelial cells by promoting cell cycle arrest in renal tubular epithelial cells, which leads to abnormal repair, activation of myofibroblasts, and production of extracellular matrix. In addition, TGF-β signaling promotes endothelial injury and myofibroblast differentiation after AKI. TGF-β, PDGF-β, and CTGF mediate fibroblast/bone marrow transformation and reinforce the endothelial-fibroblast interface that is involved in fibroblast proliferation and capillary rarefaction in the pathological process of CKD. Furthermore, TGF-β and PDGF-β are secreted by epithelial cells that fail to recover after AKI, which accelerates fibrogenesis.^10^ Future studies on the inhibition of TGF-β signaling after cessation of AKI are needed to better define the role of TGF-β in the progression of acute to chronic renal injury.^102,142^ As previously mentioned, dysregulation of VEGF signaling is a key factor in promoting renal injury in CKD, since endothelial dysfunction and failure to maintain endothelial integrity lead to renal fibrosis.^123,143^ Furthermore, many growth factors, including TGF-β, BMP-7, VEGF, and HGF, are highly involved in the course of the AKI-CKD transition through regulating inflammation and immune reactions. In this setting, targeting abnormal activation of these signals may prevent AKI progression to CKD.

## Growth factors may serve as biomarkers

To date, a series of studies have evaluated growth factors and correlated molecules as biomarkers for the early diagnosis and prediction of renal recovery from AKI. AKI diagnosis is currently dependent on increased serum creatinine (sCr) or other biomarkers. Considering that these factors are indirect biomarkers of kidney function, direct markers of tissue damage may be better candidates for predicting AKI (Table 2). As critical cell arrest modulators, the urine biomarkers IGFBP-7 and TIMP-2 are involved in the early phase of cellular stress and are used to predict AKI, especially moderate and severe AKI.^95,144^ In 2019, Kellum et al. published a guide for the clinical use of the [TIMP-2]* [IGFBP-7] biomarker to assess the risk of AKI in critical care.^145^ Recent evidence shows that these biomarkers may also predict adverse outcomes of AKI patients in the ICU.^146,147^ There are other potential biomarkers for AKI. The Translational Research Investigating Biomarker Endpoints for Acute Kidney Injury (TRIBE-AKI) cohort study on adults undergoing cardiac surgery found that proangiogenic markers, such as VEGF, correlated with a reduced risk of AKI and mortality, but antiangiogenic VEGFR-1 was associated with an increased risk of AKI and mortality.^148^ Moreover, VEGF-C and VEGF-D, which are the main ligands for lymphangiogenesis, are abundantly expressed in tubules and increased in the serum and urine after injury. They are involved in renal inflammation and possibly serve as novel urinary biomarkers for AKI and the progression of kidney disease.^149^ In addition, increased urine or plasma FGF-23 levels may be promising novel biomarkers for AKI and other adverse outcomes in critically ill patients.^148,150–152^ A previous study also showed that low IGF-1 levels might serve as mortality predictors in AKI patients in the ICU.^89^

**Table 2:** Diagnosis and treatment in patient with AKI.

	Biomarkers/novel therapy	Patients
Diagnosis	[TIMP-2]•[IGFBP7] ≤0.3 Low risk of AKI; >0.3 >92% of stage2/3 AKI; (FDA approved)^145^	Postoperative cardiovascular surgery
		Shock/hemodynamically unstable
		Sepsis
		Postoperative major non-cardiovascular surgery
		Cardiac arrest
		Oliguria after acute resuscitation
	FGF-23 (Clinical trial)	Severe sepsis/septic shock
	IGF-1^89^	In the intensive care unit
	VEGF VEGFR-1^148^	Cardiac surgery
Treatment	HGF mimetic (ANG-3777, clinical trial)	Kidney transplantation Cardiac surgery

## Growth factors and stem cell-based AKI therapy

The therapeutic effect of stem cells, especially MSCs, in AKI has been widely investigated in the last decade. MSCs can be isolated from bone marrow, umbilical cord, placenta, or adipose tissue, and they show potent anti-inflammatory and immunosuppressive properties.^153^ Previous studies found that MSC transplantation prolonged mouse survival and promoted renal repair in AKI models induced by toxic drugs and ischemic/reperfusion.^154^ Several mechanisms have been proposed regarding the effect of stem cells on renal repair, including paracrine growth factors or extracellular vesicles.^155^ Stem cells accelerate renal repair by paracrine signaling through multiple types of growth factors, such as VEGF, FGF-2, IGF, and HGF.^156,157^ However, recent studies have indicated that extracellular vesicles (EVs), particularly microvesicles and exosomes, are responsible for the therapeutic effect of MSCs in many types of disease.^158^ A previous study on the biodistribution of MSC-derived extracellular vesicles in an AKI model showed that exosomes appear to be able to move to the injury site.^159,160^ Further evidence also indicated that horizontal transfer of IGF-1 receptor mRNA to tubular cells through MSC-derived exosomes accelerates renal repair post AKI.^161^ In addition, MSC-derived extracellular vesicles directly secrete bFGF, VEGF, IGF-1, and other proangiogenic factors,^162^ which have therapeutic effects on AKI.^12^

**Fig. 3:**
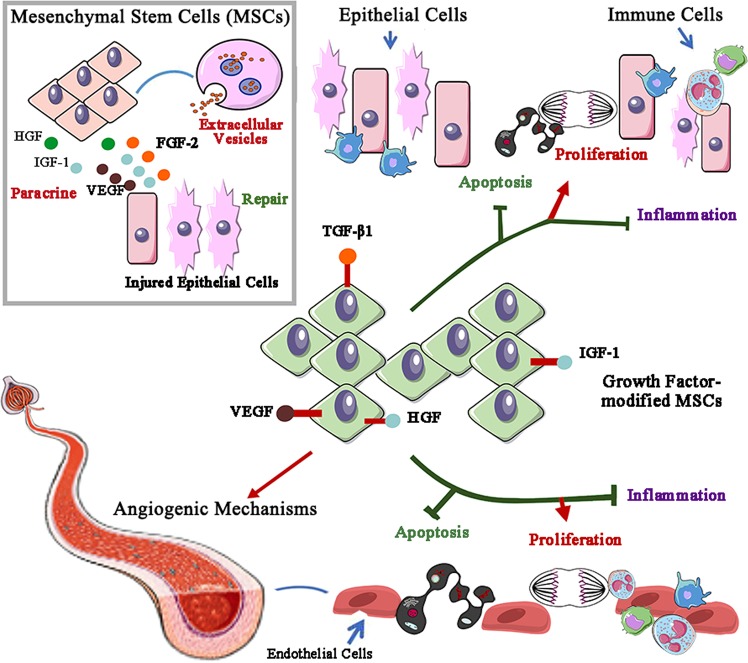
Growth factors and stem cell-based AKI therapy. Extracellular vesicle (EV)-delivered and paracrine factors such as HGF, IGF-1, VEGF, and FGF-2 from mesenchymal stem cells contribute to repair after renal injury. More importantly, stem cells modified by growth factors, including VEGF, TGF-β1, and IGF-1, efficiently protect against AKI by decreasing apoptosis and the inflammatory response and promoting tubular epithelial and endothelial cell proliferation. VEGF-modified stem cells change capillary density via angiogenic mechanisms to attenuate renal ischemia-reperfusion injury.

Moreover, growth factor-modified stem cells show more therapeutic potential than untreated controls. For example, IGF-1-incubated umbilical cord-derived MSCs had an enhanced renoprotective effect in the treatment of gentamicin-induced AKI.^163^ Consistently, a compound containing the C domain peptide of IGF-1 and chitosan hydrogel imitated the microenvironment of adipose-derived MSCs and had therapeutic effects on AKI.^164^ In addition, the VEGF165 gene conferred MSCs with protection against cisplatin-induced AKI by exerting beneficial effects on cell apoptosis, proliferation, and peritubular capillaries.^165^ In contrast, knockdown of VEGF in MSCs largely reduced the therapeutic potential of these cells and decreased the microvessel density in an AKI model.^166^ Consistently, a recent study identified that VEGF overexpression in amniotic fluid stem cells attenuated renal ischemia-reperfusion injury via mitogenic, anti-inflammatory, and angiogenic mechanisms.^167^ As a key immunomodulatory growth factor, TGF-β1-modified MSCs produce a local immunosuppressive effect and prevent IRI.^168^ Additionally, other studies indicated that HGF gene therapy or HGF-modified MSCs play a more effective role in AKI via antiapoptotic and anti-inflammatory mechanisms.^66^ A brief summary of stem cell-based AKI therapy is provided in Fig. 3.

## Anti-AKI therapy targeting growth factors

### Potential growth factor-targeted therapy for AKI

As previously mentioned, therapeutic strategies targeting growth factors and downstream effectors have been tested in animal models of AKI. BMP-7 seems to be a potential therapeutic target, since treatment with recombinant BMP-7 preserved kidney function and increased the survival rate post ischemic AKI,^17^ and restoration of BMP-7 by Dex or TSA attenuated renal injury by inhibiting HDAC5 or HDAC2-mediated suppression of BMP-7, respectively.^24^ In addition, members of the FGF family, such as FGF-10 and FGF-21, protect against AKI induced by cisplatin and IRI.^57,58^ HGF and c-met are also ideal targets because activation of HGF/c-met signaling attenuates tubular injury and renal inflammation in murine models of multiple types of AKI.^74^ Moreover, TGF-β/Smad signaling may also be a good target in the treatment of AKI because recent studies showed that the restoration of Smad7 or knockdown of Smad3 prevents both AKI and CKD progression.^118^ Although administration of VEGF effectively alleviated renal injury, we should be cautious because overactivation of VEGF in the late stage of AKI may promote the AKI-CKD transition.^123^ These strategies should be further evaluated in more animal model studies before clinical trials.

### Clinical trials of growth factors for treating AKI

Effective and specific therapies for AKI in the clinic are still unavailable, and only a small number of agents targeting growth factors have been tested in clinical trials (Table 2). A small molecule hepatocyte growth factor/scatter factor (HGF/SF) mimetic, termed ANG-3777 or BB_3_, is undergoing clinical trial in patients who are susceptible to kidney injury.^169^ Investigators from Angion Biomedica Corp have demonstrated that ANG-3777 improves renal function in patients after kidney transplantation.^170^ Furthermore, research by this company is assessing whether ANG-3777 can reduce the severity of delayed graft function in recipients of a deceased donor kidney.^171^ Other similar clinical trials are underway. A phase 2 study to assess the safety and efficacy of ANG-3777 in patients who develop AKI after cardiac surgery is ongoing.^172^ Unfortunately, some clinical trials have already failed; for example, exogenous IGF-1 is beneficial in the recovery after kidney injury in mouse models, but a therapeutic trial in patients with acute renal failure (ARF) failed to demonstrate the efficacy of IGF-1 in humans because it induced a fibrotic response in mesangial cells and extensive neutrophil infiltration that reduced patient survival.^56^ This may be due to different renal lesions in ARF. Patients with ARF always have other severe illnesses, unlike experimental models with isolated disorders. Taken together, more precise dosing and targeted drug delivery systems need to be used and further studied.

## Concluding remarks

In conclusion, growth factors function in the entire process of AKI, including initiation, renal repair, and the AKI-CKD transition. Considering the multiple roles of growth factors in kidney injury, directly targeting them may result in unexpected side effects such as renal fibrosis, which may impede their clinical application. Therefore, their downstream effectors should be characterized and evaluated as new targets in future studies. In addition, growth factors and correlated proteins, such as IGFBP-7, could serve as biomarkers for the prediction of AKI. MSCs modified by certain growth factors have great merit and may contribute to AKI treatment in the future.
